# Barriers and Facilitators to Coping with Second Victim Experiences: Insights from Nurses and Nurse Managers

**DOI:** 10.1155/2024/5523579

**Published:** 2024-08-02

**Authors:** Xizhao Li, Mei-Chan Chong, Chong-Chin Che, Yamin Li, Ling Wang, Alan Dong, Ziqing Zhong

**Affiliations:** ^1^ Department of Nursing Science Faculty of Medicine University of Malaya, Kuala Lumpur, Malaysia; ^2^ Clinical Nursing Teaching and Research Section The Second Xiangya Hospital Central South University, Changsha, China; ^3^ Florence Nightingale Faculty of Nursing Midwifery and Palliative Care King's College London, London, UK

## Abstract

**Background:**

Second victim experiences have long-term impacts on the personal and professional well-being of nurses. Individual-centered support is necessary to help nurses cope with the various stages of the second victim experience.

**Objectives:**

To explore personal and workplace factors that facilitate or hinder coping styles for second victim experiences from the perspectives of both frontline nurses and nurse managers.

**Design:**

This was a descriptive qualitative study that incorporated semistructured interviews.

**Methods:**

Purposive sampling was employed to enlist a total of eight nurses and seven nurse managers selected from five tertiary hospitals located in Hunan Province, China. The study participants included nurses who had suffered second victim experiences and nurse managers who had grappled with their nurses' second victim experiences. The data were transcribed verbatim and analysed using thematic analysis.

**Results:**

The analysis revealed four main themes that influenced nurses' ability to cope with second victim experiences: source of emotional trauma, personal factors, job stress, and support system. In contrast, emotional trauma from patients and relatives, negative personal traits, shadows from the second victim experience, and unsupportive workplace environments were obstacles to coping with second victim experiences.

**Conclusion:**

The study highlights facilitators and barriers that nurses cope with second victim experiences, providing insight to develop targeted interventions that support nurses and mitigate the negative impacts of second victim experiences. A comprehensive approach is more effective in supporting nurses in coping with second victim experiences, improving patient safety, and enhancing the quality of care.

## 1. Introduction

According to the Lancet Global Health Commission, patient safety issues remain a critical challenge worldwide, with 134 million adverse events occurring annually in low- and middle-income countries [1]. In China, adverse event reporting is a crucial criterion for evaluating patient safety in hospitals, but it often lacks effective support systems for nurses, resulting in punishment and isolation [2, 3]. Despite the continuing challenge to nurses of an unprecedentedly burdened, complex, and ever-changing work environment [4], the specific personal and workplace factors that arise during adverse events are still unknown.

Over the past two decades, nurses encountering adverse events have been characterized as “second victims.” This term describes nurses who undergo emotional and psychological distress following such events, which can significantly impact their well-being and professional performance [5, 6]. Studies have indicated that more than two-thirds of nurses involved in second victim experiences troubling memories, anxiety, anger, and distress. Troubling memories were found to be the most prevalent symptom, with a frequency of 81% [5]. The most commonly reported symptom among nurses involved in second victim experiences was hypervigilance, and doubts about their skills and knowledge were also reported frequently [7]. Furthermore, the research found that the symptoms of second victim experiences were more enduring and severe among nurses than physicians [8]. The COVID-19 pandemic exacerbated the stress and anxiety faced by nurses, including challenges to their health [9]. As reported, empowering nurses with confidence, tools, and skills is critical to improving patient safety and could result in 1,000 lives saved each year by 2024 [10]. Gaining insight into the obstacles and requirements faced by nurses impacted by second victim experiences is crucial for aiding their recovery and progression [11].

Additionally, there is a lack of evidence on effective coping styles for nurses in second victim experiences. Coping refers to the thoughts and behaviours that individuals use to manage the internal and external demands of stressful events [12]. Several studies have highlighted that nurses may struggle with effective coping with second victim experiences due to poor awareness and delayed access to support programmes. For example, Edrees et al. [13] found that delays in receiving support programmes were a theme related to coping with second victim experience among nurses. Similarly, previous studies reported that seeking support and forgiveness from peers and managers was a common coping strategy among nurses [14, 15]. However, there is a lack of literature that provides a comprehensive understanding of the specific personal and workplace factors that influence coping among nurses in second victim experiences, highlighting the need for further research. Moreover, a recent study by Steven et al. [16] highlights the importance of coping as a significant professional practice for nurses in managing emotional and relational tension in clinical settings. However, existing literature has only briefly addressed the tendency to delay accessing support programmes in second victim experience, which can lead to a worse professional quality of life and negative consequences for patient safety [17, 18]. While a mixed methods systematic review by Pollock et al. [19] found that awareness of frontline workers' needs can facilitate mental health support, minimal research has focused specifically on the coping process of second victim experiences, especially from the perspectives of nurses and nurse leaders. Studies have shown that nurse managers play a crucial role in maintaining a creative work environment and the well-being of patients and nurses, but constraints such as time, workload, and resources can lead to poor communication, condescending attitudes, and bullying towards nurses [20, 21]. Moreover, nurse managers themselves can also experience negative effects when supporting nurses involved in second victim experiences [13], underscoring the challenges nurse managers face in maintaining their well-being while assisting their staff. It is therefore presumed that the well-being of nurse managers and their experiences of stress and emotional challenges may exacerbate the causes of poor mental health among nurses.

This study results emphasize the significance of comprehending the factors that influence coping strategies in nurses who undergo second victim experiences, taking into account the viewpoints of both the nurses and their nurse manager. This research centers on identifying the barriers and facilitators that shape coping styles among nurses who have endured second victim experiences. Additionally, it aims to offer empirically grounded insights that can aid in crafting effective coping styles. These styles are designed to equip and support nurses in managing the challenges associated with second victim experiences.

## 2. Method

### 2.1. Study Design

This study utilised a descriptive, qualitative approach through individual face-to-face semistructured interviews. The study recruited nurses as participants from tertiary care hospitals using purposive sampling, while nurse managers were identified through snowball sampling. This process continued until data saturation was reached, indicating that no new information could emerge from the data. [22]. The data collected were analysed using Braun and Clarke's [23] method of thematic analysis in NVivo Version 12, based in Lumivero, USA. The researchers followed the report guidelines of consolidated criteria for reporting qualitative research [24]. To prevent any unintended impact from relationship dynamics, there were no pre-existing relationships among the researchers, nurses, or nurse managers involved in the study.

### 2.2. Participants

The research was carried out in five tertiary hospitals in Hunan Province. Nurses and nurse managers who had previously participated in an online survey regarding second victim experiences and agreed to share their insights were involved in this study [25]. Furthermore, nurse managers were enlisted through snowball sampling, leveraging referrals from participating nurses and existing nurse managers within the study. The criteria for selecting nurses included the following: (1) had second victim experience and (2) at least one year of being employed full-time. For nurse managers, the criteria were as follows: (1) holding a pivotal leadership role within the hospital's nursing team, tasked with overseeing nursing operations in a medical unit, which encompasses planning work schedules, supervising the implementation of nursing care, mentoring junior nurses, and facilitating communication between doctors and patients and (2) having experience in providing support to nurses who have faced second victim experiences. The researcher (XZ) contacted and invited participants who had completed an online survey on second victim experience between 2021 and 2022. A total of 15 participants, comprising 8 nurses and 7 nurse leaders, were interviewed individually to obtain their perspectives.

### 2.3. Research Team Characteristics

The research team was exclusively female and consisted of three nurses (XZ, LW, and ZQ), two nurse managers (YM and AL), and two nursing science lecturers (CMC and CCC). Nurse XZ was a PhD candidate at the University of Malaya, LW was a PhD candidate at King's College London, and ZQ was a master's student at Central South University. Three team members (CMC, CCC, and YM) hold a PhD qualification and have expertise in qualitative research. The nursing science lecturer CMC, an Associate Professor at the University of Malaya, has extensive experience in qualitative analysis and is not affiliated with the hospital to avoid any potential impact from relationship dynamics.

### 2.4. Data Collection

Data were collected between February 2022 and August 2022. Participants were recruited through an advertisement on a WeChat page that provided information about the study, and interested individuals were screened for eligibility criteria before providing consent to participate. The interviews were conducted by XZ at a mutually convenient time and location for the participants. Nurses LW and ZQ were the field note-takers, with LW joining the interviews with participants identified as RN01–08 and ZQ joining the interviews with participants identified as NL01–07. Each interview lasted between 40 and 60 minutes and was audio recorded. The interviews took place in the medical unit's designated rest area, which is furnished with a comfortable sofa and provides a quiet atmosphere. Participants were able to book the rest area for at least an hour and had the option to lock the door during the interview for privacy. The interviews were scheduled for either noon or after the participants' duty hours to ensure their availability. The interviews aimed to obtain insights into the barriers and facilitators of coping with second victim experiences among nurses. Demographic data were collected, including the gender, age, length of working experience, position, title, medical unit, and education level. The interview guide was developed by the authors (XZ and LW) based on the previous literature and is presented in Table 1. To ensure consistency, all interviews were conducted by XZ. Data collection was considered completed when data saturation was achieved. Audio recordings of the interviews were transcribed verbatim immediately.

### 2.5. Data Analysis

This study followed the six-step thematic analysis method developed by Braun and Clarke [23] and utilised NVivo v12 to manage and analyse the qualitative data. The data were transcribed and anonymised before being coded by XZ and LW independently for each participant. The first step involved familiarising the researchers with the data sets and generating initial codes. To ensure completeness, the coded data were compared with the transcripts and returned to the participants; there are no comments and corrections from the participants. The third step involved identifying potential themes, which were reviewed and refined during the fourth step. Several rounds of discussion were conducted to ensure the representation, consistency, and accuracy of the themes. The refined themes were then converged to ensure consistency and accuracy. The fifth and sixth steps involved defining and naming the themes and presenting thematically the results of the study. The thematic presentation aimed to provide an in-depth understanding of the facilitators and barriers that nurses face when coping with second victim experiences. The rigorous analysis approach helped to ensure the trustworthiness and reliability of the findings.

### 2.6. Rigour and Trustworthiness

To increase the credibility of the findings, this study used member checking and peer debriefing. Member checking involved presenting the research findings to participants to confirm accuracy and receive feedback, while peer debriefing involved other researchers reviewing the study design, data collection, and analysis.

Triangulation was used to increase the understanding of the phenomenon. Triangulation is important when studying frontline nurses and nurse managers because it recognises the differences in their roles and responsibilities. These roles often result in different understandings, priorities, and perspectives between the two groups. Moreover, this approach entails gathering and analysing data from various sources, encompassing both interviews and observations. Observational methods enable researchers to capture data that participants might not readily convey in interviews, thereby offering a more comprehensive perspective on the phenomena being investigated. This holistic approach aids in fostering a dependable understanding of perceptions regarding the second victim experience. This triangulation approach significantly increases the validity and rigour of the research findings [26].

The utilization of observation notes during data collection involves three primary stages, encompassing both interviews and observational methods. Initially, after transcription, observation notes are amalgamated with interview transcripts. These annotated records, serving as observational insights, are seamlessly integrated into the transcribed manuscripts, enriching the initial coding phase with a comprehensive perspective. Subsequently, the transcribed manuscripts and observation notes undergo review with research participants to elucidate any potential ambiguities in conveyed meanings. Once themes are established, they undergo further examination in conjunction with observation notes to validate and refine emerging patterns. The incorporation of observational data throughout this methodology significantly enhances thematic analysis, fostering a deeper understanding and more nuanced insights. Post-transcription, the integration of observations with interview data affirms the coherence and expansion of emerging themes across diverse data sources.

The researchers (XZ and LW) compared the information gathered from each source. By including the perspectives of both frontline nurses and nurse managers, this study was able to gather a complete understanding of the facilitators and barriers to coping with second victim experiences. In addition, member checking was used as a verification technique upon the full development of themes, thereby bolstering the validity of the research findings.

### 2.7. Ethical Consideration

Before conducting this study, ethical approval was obtained from the Research Ethics Committee at the Second Xiangya Hospital of Central South University (no. LYF2022003). All participants were provided with research statements, and informed consent was obtained, with opportunities for questions and the right to withdraw from the study at any time. This study was conducted following ethical principles and guidelines for research involving human subjects. Participants who experienced psychological distress and wished to receive help would be evaluated by project team members and provided with assistance. If any discomfort arose during the interview process, participants could contact a mental health professional from the Department of Psychiatry at Xiangya Second Hospital, Central South University, at 0731-85295555, if necessary.

## 3. Results

### 3.1. Demographic Characteristics of Participants

Table 2 presents the demographic characteristics of the 15 participants in this study. The majority of the study's participants were female, comprising 93% of the sample, while male participants constituted a smaller portion at 7%. The average age of the participants was 38.9 years, and they had an average professional experience of 16.7 years. Nearly half of the participants (47%) held advanced professional titles, and 40% had intermediate titles. The most represented medical units were internal medicine and surgery, each contributing a third of the participants, followed by a smaller percentage in specialties such as paediatrics, obstetrics, and gynaecology, which accounted for 13%. In terms of educational background, 20% of the participants, amounting to 3 individuals, possessed a bachelor's degree, whereas the majority, 80% or 12 participants, held a master's degree.

### 3.2. Themes and Subthemes

This study aimed to explore personal and workplace factors that facilitate or hinder coping styles among nurses who have second victim experience. It employed a thematic analysis of semistructured interview transcripts, which resulted in 4 main themes and 12 subthemes (Table 3). The 4 main themes that emerged from the analysis were the source of emotional trauma, personal factors, workplace environment, and support systems. Each theme was further divided into subthemes, providing more specific information from both nurse and nurse manager perspectives.

#### 3.2.1. Source of Emotional Trauma

In the second victim experience, nurses are exposed to trauma from different sources that can impact their coping styles.

*(1) Patients' Consequences*. When patients experience adverse consequences, nurses often internalise a sense of responsibility, leading to guilt and self-blame. This emotional burden can intensify feelings of stress and anxiety and impede their ability to cope effectively.

*NL02: Their blood pressure is too high! and then, er… the patient told me, uncomfortable and crappy, you know what I mean? It's so dangerous…If it is not dealt with in time, the consequences will be disastrous*.

*(2) Response of Patients' Relatives*. Patients' relatives can respond in various ways, including hostility, blame, denial, or lack of cooperation. These responses can increase the nurse's stress and emotional distress, leading to feelings of anger, resentment, and burnout.

*NL05: The family, in fact, had similar views to his; that is, the family couldn't understand, and was also very anxious, er… like can't listen to the explanation, very angry.*

#### 3.2.2. Personal Factors

This refers to individual traits, experiences, abilities, and attributes that may influence how a nurse copes with the second victim experience.

*(1) Personality*. Sensitivity may lead to increased emotional distress. However, positive thinking may help nurses find meaning and purpose in their work, contributing to their well-being.

*RN07: Having worked for over ten years, and I'm a relatively sensitive and fragile person, the way I deal with this, always be impressive in my mind.*

*RN05: I was shocked, and I have learned a lesson from this incident, so I will not handle this pipeline problem recklessly again. In the future, I will take the initiative to ask the seniors to look at any problem that I think I am not sure about or any problem of this kind.*

*(2) Shadows of Experience*. It highlights personal factors following adverse events that affect how nurses cope with the second victim experience, referring to the emotional, physical, occupational, and long-term consequences that negatively affect coping styles.

Emotional echoes: Nurses experiencing anxiety and fear lead to avoidance coping styles, while those dealing with guilt might adopt more conflictual coping styles.

*RN07: After the incident, but er… There was, at the time, a lot of nerves…and when I'm wrong, it's really aggravating, scary and helpless.*

Physical reactions: Physical discomfort or illness may limit the energy and resources available for coping, leading to a preference for less active or avoidance-based styles.

*RN04: Just sitting on the floor, crying ah. Sometimes, sometimes sitting on the bed without moving, can really sit for 12 hours without eating or drinking.*

Occupational challenges: To highlight how adverse events can damage nurses' confidence in their clinical skills and decision making, impact their professional identity, and lead to negative coping styles as a result of avoidance.

*NL03: Eventually, for a while, I did notice that she was kind of wandering around at work. Then she said she wasn't right for the position… She mentioned resigning later. She wrote her resignation letter anyway.*

Enduring silence: It suggests that nurses' struggle with unresolved emotions without effective processing may exacerbate chronic worrying and rumination, thereby influencing the adoption of negative coping styles.

*RN05: It was about, over 10 years ago, and I am still so sad, every time I mentioned it, oh my God….*

*NL02: But, that long process is really completely insomnia, that is the bad event for her to cause this kind of damage, this event for her this life is really to be engraved in the heart, afraid to mention to anyone to talk about.*

#### 3.2.3. Job Stress

The physical and psychological conditions of the work setting can affect nurses' response to the second victim experience, including organizational culture, job demands, and workload.

*(1) Workload*. It refers that the high level of workload led to avoiding the stressor.

*RN01: Some time ago, because of the lack of manpower and the seriousness of the patient's condition, during the New Year, there were more unplanned extubating, for example, It seems that we can't change anything, out of control, hard to believe…*

*(2) Ineffective Management*. This refers to a situation where the management fails to provide adequate resources, support, and guidance to nurses in dealing with the second victim experience.

*RN01: To report adverse events, you must do courseware and PPT, and all nurses involved should report… It will increase the workload, and everyone will be very stressful…*

*(3) Organisational Culture Barriers*. Cultural barriers within a healthcare organisation can hinder the ability of nurses to manage the second victim experience effectively.

Inadequate awareness: Insufficient knowledge or comprehension of the second victim experience escalates the risk and hampers the capacity to cope effectively.

*NL03: Regardless of whether there are consequences or not, it really feels like an adverse event, and an adverse event is that I did not do well…*

Punishment effects: Punishing nurses without a supportive environment can lead them to develop unhealthy ways of coping with the stress and emotional impact of second victim experiences.

*RN03: In fact, at that time, I have the feeling that there are no set rules and regulations when it comes to punishment; it's more like decisions are made on the basis of the thoughts of the heads of medical units or nursing units, without a clear basis…*

#### 3.2.4. Support Systems

Support systems can play a critical role in how nurses cope with the second victim experience, and they refer to networks of individuals, groups, and organizations that provide emotional, practical, and informational support to nurses.

*(1) Colleague Network*. A colleague network for nurses refers to a group of colleagues who can provide support to the nurse in the second victim experience, including practical advice and emotional support.

*RN07: The support of the nurse managers and the doctor, for example, the doctor went to help the patient, and after explaining and apologising together, the attitude of the patient eased up. My, this emotion, this nervous anxiety, this helpless emotion, There is a noticeable decline.*

*(2) Continuous Professional Education*. This refers to ongoing education and training on skills and knowledge related to reviewing adverse events. Such education can help nurses gain a better understanding of the second victim experience and how it can be prevented. It includes understanding the signs of mental health conditions, knowing how to seek help, and recognising the importance of wellness.

*RN04: I hope he can guide me in the future, when I encounter such accompanying family members, guide nurses on how we should skilfully resolve this conflict.*

*NL05: You analyse it, you analyse it all, you do it all, and the next time he'll know how, yeah… How to understand this matter. I'm going to handle it, how I'm going to do it. I'm not going to be passive, and I'm going to be proactive… Especially, I can accept myself as a nurse, and I also need to seek help.*

*(3) Availability of Referral Resources*. This refers to the presence of adequate resources that can assist the nurses in effectively coping with second victim experiences, such as time and mental health services.

*NL03: During the rest time, we would accompany her to see a doctor later… After talking with the director, I also talked to a few members of our nursing core team about this matter, for protecting…and helping her.*

*(4) Family and Community Support*. This refers to the role that family members and the community can play in supporting nurses who are involved in the second victim experience.

*RN08: But in the end, I still face it by myself. Of course, my family has always supported me. Even if they don't study medicine, they are not very capable. They are very clear about the whole process, but they will accompany me.*

## 4. Discussion

The findings of this study underscore crucial elements that can either hinder or aid nurses in dealing with second victim experiences. These factors play a significant role in influencing how nurses manage and recover from such challenging situations. Although qualitative studies from various countries, such as Australia, Switzerland, and the United Kingdom, highlight the significance of individual-centered coping styles that are based on the different stages of the second victim experience [11, 27, 28], there is still a lack of in-depth evidence on the factors that impact these coping styles. This study considered the perspectives of both nurses and nurse managers and identified several dimensions that play a role in the coping process. The results of this study indicate that coping with second victim experiences is shaped by emotional trauma origins and personal traits. Additionally, the study uncovers that workplace-related challenges significantly add to the stress of second victim experiences, consequently impeding the efficacy of the coping process for nurses. Conversely, a multilevel support system is unanimously recognized by both frontline nurses and nurse managers as a critical facilitator in managing second victim experiences. This comprehensive support structure plays a pivotal role in aiding nurses to effectively navigate and cope with the challenges arising from these experiences.

The theme of the source of emotional trauma in this study revealed a complex association between patients' outcomes, relatives' responses, and the second victim experience among nurses. It emphasizes that patients' consequences directly impact the nurses, aligning with studies indicating that nurses can feel responsible for patient consequences and fear disciplinary action, leading to negative coping strategies such as avoidance, denial, or self-blame [6]. Researchers have identified marked disparities in how adverse events are perceived by nurses, patients, and their families [29, 30]. Additionally, difficulties in the interactions between nurses and patients' relatives have been documented in a qualitative meta-analysis [31]. This study further emphasizes the deep-seated connection between patients' relatives and nurses who have gone through second victim experiences. The participants of this study shared that relatives often undergo feelings of anger, grief, and loss after adverse events, and these emotions are frequently projected onto the involved nurses. In some cases, this can escalate to legal actions, which intensify the nurses' challenge in coping with their second victim experiences. These observations underscore the necessity for healthcare organizations to enhance communication and coordination following adverse events [32, 33]. Implementing standardized procedures that enable nurses to effectively communicate about adverse events and ensure patients' families have a clear understanding of what occurred can help mitigate blame and conflict. Such measures could significantly improve nurses' ability to manage and recover from second victim experiences.

This study has pinpointed several personal factors such as personality and overall damages from adverse events as crucial determinants influencing the coping styles of nurses. To begin with, personality traits have a significant impact on how nurses handle second victim experiences [34]. It has been observed that participants who exhibit positive thinking are generally more adept at coping with these experiences. Supporting this, prior research indicates that nurses often display higher levels of extroversion compared to professionals in other fields, which can influence their coping methods [35]. However, the study also notes that nurses with high sensitivity might experience increased emotional distress when dealing with second victim experiences, indicating a nuanced relationship between personality traits and coping effectiveness. These findings align with those of previous research, which indicates that nurses can feel threatened when there is a significant disparity between their expectations and reality [36]. Importantly, some participants—three nurses and two nurse managers—revealed that even though as many as 15 years have passed since the second victim experience, the sadness, fear, and anxiety of the event still lingers as they recount their experiences. Some of the nurse managers reported long-term effects such as depression and career changes. Findings in the shadow of experience add to the growing evidence that multiple harms following adverse events significantly influence coping styles. [8, 37]. It highlights the need to break the cycle of silence and promote healing of emotional, physical, and professional dimensions, as well as ongoing reflection to recover from trauma.

The third theme in this study emphasised the challenges related to the job stress for nurses in coping with the second victim experience; in particular, it highlights the recognition of the burdens posed by the intricate nature of the prevailing management processes. Some participants of this study reported that high levels of workload cause nurses to experience higher levels of stress, leading to increased negative coping to the second victim experience. Significantly, convoluted reporting processes during adverse events can subject nurses to pressure and a lack of support within the context of the second victim experience. In turn, it can lead to deficient factor analysis and the adoption of ineffective coping styles [38]. Furthermore, the findings of this study suggest that in the absence of these supportive mechanisms, nurses may face difficulties in effectively processing their emotions and experiences. This challenge could potentially result in prolonged psychological distress, an increased risk of burnout, and could even lead to nurses leaving the profession. The importance of adequate support structures is thus underscored, as they are critical in helping nurses navigate the complexities of their experiences and maintain their mental health and career longevity. A ten-year national survey recommends the importance of fostering a supportive work environment where nurses feel valued and heard and where they can openly discuss their experiences and receive appropriate support to cope with the emotional impact of the second victim experience [39].

Theme four of this study highlights the most significant findings in facilitating coping with the second victim experience among nurses. The study discovered that having a network of colleagues is advantageous, echoing findings in the existing literature that highlight how colleague networks are integral to shaping an organization's culture and in providing a safe environment for expressing emotions. Previous research has underscored that when nurses are part of a supportive peer network, they feel more empowered to manage complex situations without fear of judgment or criticism [28]. However, an interesting point raised by participants in this study, which appears to be underrepresented in current literature, is that the most immediate and effective support often comes directly from colleagues. This aspect emphasizes the crucial role of peer support in the immediate aftermath of challenging incidents, indicating a potential area for further exploration and emphasis in future research and organizational policies. Continuous professional education is revealed in this study, as Goh et al. [40] and Davis et al. [41] supported that it is crucial for maintaining resilience, collaborative practice, and patient safety. Previous studies have similarly demonstrated a link between mental health literacy and adaptive coping styles [42, 43]. Enhancing mental health literacy among nurses can better prepare them to manage the emotional challenges associated with the second victim experience. Continuous learning and skill development can empower nurses to effectively navigate the demands and stressors of these experiences. Moreover, the findings from our study suggest that mental health literacy can also benefit nurse managers in supporting nurses during complex situations. Younas et al. [21] revealed that mental health literacy plays a crucial role in enabling nurse managers to address their emotions effectively and implement successful management strategies. The concept of the second victim remains a topic of debate, with some critics such as Tumelty [44] arguing that it might detract from the focus on patient harm. Despite these contentions, findings from this study reveal that frontline nurses often face a lack of clear guidance on how to seek help for second victim experiences, which can lead to the adoption of negative coping mechanisms. On the other hand, nurse managers are in favor of creating a structured referral system. Such a system, as suggested by Norvell et al. [45], would be designed to swiftly identify nurses experiencing second victim phenomena and provide them with appropriate mental health services. This approach could potentially bridge the gap between the immediate needs of affected nurses and the long-term goal of maintaining high standards of patient care, while also addressing concerns about the potential overshadowing of patient harm. This can ensure that nurses receive timely and appropriate care and support to cope with the emotional and psychological effects of their experiences. In Chinese culture, both family and community constitute fundamental pillars of an individual's support system [46, 47]. In addition, it is imperative to recognise that nurse managers, who simultaneously occupy family roles as mothers, wives, and daughters, play a central role in providing immediate support to fellow nurses. Consequently, the findings underscore the importance of a robust support system, including both family and community, that not only assists nurses with guilt and anxiety but also provides nurse managers with an essential resource that facilitates their prompt and effective support of nursing staff.

### 4.1. Strengths and Limitations

The strengths of this study are its rigorous qualitative design and triangulation. Frontline nurses are the ones directly involved in the second victim experience, while nurse managers have a broader view of the organizational context. By including both perspectives, this study demonstrates that coping with second victim experiences is not only a personal issue. It is also influenced by organizational and managerial factors.

Limitations include potential selection bias from different experience timeframes and gender bias. This highlights the need for ongoing research and interventions to support nurses in coping with second victim experiences.

## 5. Conclusion

The findings from this study strengthen the understanding of facilitators and hurdles in managing second victim experiences as reported by frontline nurses and nurse managers. It highlights the critical need for a comprehensive approach involving patients, their families, and healthcare institutions. By incorporating an understanding of multiple factors influencing dealing with the second victim experience, we can develop interventions that address multiple aspects to support effective coping styles among nurses.

## Figures and Tables

**Table 1:** Interview guide.

(1) What are some of the most challenging adverse events you (your colleagues) have experienced in your career?
(2) How did it affect you (your colleagues)?
(3) How did you (your colleagues) cope with the events?
(4) How did the organisation manager manage the events?
(5) How did interactions between you and your team influence your experiences?

**Table 2:** Demographic details of participants (*N* = 15).

Variable	Category	%(*N*)/mean ± SD
Gender	Female	14 (93)
	Male	1 (7)

Age (years)		38.9 ± 8.5

Years of working		16.7 ± 9.1

Title	Junior	2 (13)
	Intermediate grade	6 (40)
	Advanced title	7 (47)

Medical unit	Internal medicine	5 (33)
	Surgery	5 (33)
	Paediatrics, obstetrics, and gynaecology	2 (13)
	Acute and intensive care	3 (20)

Education level	Bachelor	3 (20)
	Master	12 (80)

**Table 3:** Themes and subthemes of the study.

Themes	Subthemes
Theme 1. Source of emotional trauma	1.1. Patients' consequence
	1.2. Response of patients' relatives

Theme 2. Personal factors	2.1. Personality
	2.2. Shadows of experience
	2.2.1. Emotional echoes
	2.2.2. Physical reaction
	2.2.3. Occupational challenges
	2.2.4. Enduring silence

Theme 3. Job stress	3.1. Workload
	3.2. Ineffective management
	3.3. Organisational culture barriers
	3.3.1. Inadequate awareness
	3.3.2. Punishment effects

Theme 4. Support system	4.1. Supportive network of colleagues
	4.2. Continuous professional development
	4.3. Availability of referral resources
	4.4. Family and community support

## Data Availability

The datasets generated during and/or analysed during the current study will be shared only on request with the approval from the Universiti Malaya.
